# Antimicrobial activities of the methanol extract and compounds from *Artocarpus communis *(Moraceae)

**DOI:** 10.1186/1472-6882-11-42

**Published:** 2011-05-25

**Authors:** Victor Kuete, Patrick Y Ango, Ghislain W Fotso, Gilbert DWF Kapche, Jean P Dzoyem, Arlette G Wouking, Bonaventure T Ngadjui, Berhanu M Abegaz

**Affiliations:** 1 Department of Biochemistry, Faculty of Science, University of Dschang, Cameroon; 2 Department of Chemistry, Higher Teachers' Training College, University of Yaoundé I, Cameroon; 3 Department of Organic Chemistry, Faculty of Science, University of Yaoundé I, P.O. Box 812, Yaoundé, Cameroon; 4 Department of Chemistry, Faculty of Science, University of Botswana, Private Bag 00704, Gaborone, Botswana

## Abstract

**Background:**

*Artocarpus communis *is used traditionally in Cameroon to treat several ailments, including infectious and associated diseases. This work was therefore designed to investigate the antimicrobial activities of the methanol extract (ACB) and compounds isolated from the bark of this plant, namely peruvianursenyl acetate C (**1**), α-amyrenol or viminalol (**2**), artonin E (**4**) and 2-[(3,5-dihydroxy)-(*Z*)-4-(3-methylbut-1-enyl)phenyl]benzofuran-6-ol (**5**).

**Methods:**

The liquid microdilution assay was used in the determination of the minimal inhibitory concentration (MIC) and the minimal microbicidal concentration (MMC), against seven bacterial and one fungal species.

**Results:**

The MIC results indicated that ACB as well as compounds **4 **and **5 **were able to prevent the growth of all tested microbial species. All other compounds showed selective activities. The lowest MIC value of 64 μg/ml for the crude extract was recorded on *Staphylococcus aureus *ATCC 25922 and *Escherichia coli *ATCC 8739. The corresponding value of 32 μg/ml was recorded with compounds **4 **and **5 **on *Pseudomonas aeruginosa *PA01 and compound **5 **on *E. coli *ATCC 8739, their inhibition effect on *P. aeruginosa *PA01 being more than that of chloramphenicol used as reference antibiotic.

**Conclusion:**

The overall results of this study provided supportive data for the use of *A. communis *as well as some of its constituents for the treatment of infections associated with the studied microorganisms.

## Background

*Artocarpus comminis *J.R. & G. Forst., commonly known as breadfruit tree because of the "bread-like texture" of its edible fruits, is an equatorial lowland species of flowering tree in the mulberry family (Moraceae) that grows best below elevations of 650 m [1]. Numbers of medicinal uses are assigned to plants of the genus *Artocarpus *worldwide. This includes treatments of cardiovascular diseases (yellow leaf decoction of *A. communis *in Bahamas, Haiti, Trinidad and West Indies), chest pain and vomiting from heart problems (*Artocarpus *spp. in South Pacific), boils, abscess, and skin infections (leaf ash, macerated root, or latex of *Artocarpus *spp. sap in Dominican Republic, Haiti, Hawai'i, Malaya, Java, Samoa, Tahiti and Tonga), cracked-skin and dermatosis (*A. communis *in Hawai'i), burns (*A. communis *in Haiti), rashes **(**sap of *Artocarpus *spp. in Tahiti, Tonga); stomach pain (bark of *Artocarpus *spp. diluted latex in Samoa, Solomon Islands and Tonga), diarrhea or dysentery (diluted latex or roots boiled of *Artocarpus *spp. in Borneo, Java, Pacific Islands and Samoa), diabetes (yellow leaf as tea of *Artocarpus *spp. in Trinidad, West Indies), headache (leaves of *A. communis *in Bahamas, bark in Samoa and Pacific Islands, toothache (toasted flowers of *A. communis *and *A. integra *in Java and Malaya), thrush (crushed leaf buds and latex of *A. communis *on tongue in Bahamas, Trinidad and Pacific Islands), eye problems (*A. communis *leaf or petiole juice in Futuna and Samoa), ear infections (leaves juice or diluted latex in Pacific Islands), herpes infections (*A. communis *in Amboina), fever (*A. communis *leaves in Bahamas, Malaya and Samoa), enlarged spleen (*A. communis *in Java) [2]. In Cameroon, the fruits of *A. communis *are used as food; other parts of the plants are traditionally used to treat headache, infectious and associated diseases such as toothache, eye problems, ear infections, herpes, enlarged spleen, sprains, contusions, swelling [3-5]. Some scientific evidences of the bioactivity of *A. communis *were reported on the extract or isolated compounds [6-9]. However, few reports are related to the antimicrobial activity of this taxon. The present work was therefore designed to investigate the antibacterial and anticandicidal activities of the methanol extract and compounds isolated from the stem bark of *Artocarpus communis*.

## Methods

### Plant material

The roots of *Artocarpus communis *J.R. & G. Forst. were collected in Nkolbisson, Center region of Cameroon in March 2010. The plant was identified by Mr. Victor Nana of the National herbarium (Yaoundé, Cameroon) where a voucher specimen was deposited under the reference number 43982/HNC.

### Extraction and purification

**Figure 1:**
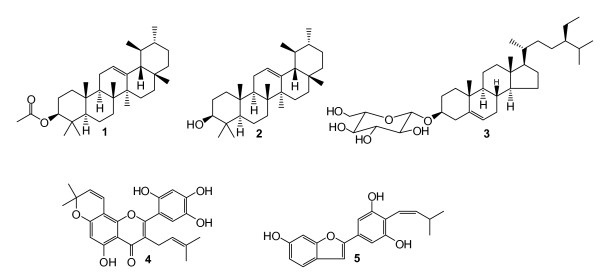
**Chemical structures of compounds isolated from the bark of *Artocarpus communis***. **1**: peruvianursenyl acetate C; **2**: α-amyrenol or viminalol; **3**: sitosterol 3-O- *ß-D*-glucopyranoside; **4**: artonin E; **5**: 2-[(3,5-dihydroxy)-(Z)-4-(3-methylbut-1-enyl)phenyl]benzofuran-6-ol.

The air dried and powdered stem bark (700 g) were extracted with methanol (MeOH) for 48 h at room temperature. The extract was then concentrated under reduced pressure to give 170 g of a brown residue that constituted the crude extract (ACB). Part of FPR (150 g) was submitted to silica gel 60 (0.04-0.063 mm, 120 g) vacuum column chromatography using as eluent, hexane, hexane/CHCl_3 _1:1 mixture, CHCl_3 _and CHCl_3_/MeOH. Fractions of 500 ml each were collected, concentrated under vacuum and pooled on the basis of the thin layer chromatography (TLC) analysis in six fractions, A-F. Peruvianursenyl acetate C_32_H_52_O_2 _(**1**; 4.4 mg; m/z 468.40; amorphous powder) [10] and α-amyrenol C_30_H_50_O (**2**; 70.3 mg; m/z 426.99; amorphous powder) [11] were directly obtained from fractions eluted with hexane/CHCl_3 _7:3. Sitosterol 3-O- *ß-D*-glucopyranoside C_17_H_18_O_4 _(**3**; 5.5 mg; m/z 286.12; whitish powder, m.p. 130-145°C) [12] was directly obtained from fractions eluted with CHCl_3_/MeOH 8:2. Fraction E (10.0 g) obtained with CHCl_3 _was subjected to CC (silica gel 60, 50 g) and eluted with CHCl_3_-MeOH mixture of increasing polarity to give 7 sub-fractions (E_1_-E_7_). Sub-fraction E_1 _(CHCl_3 _to CHCl_3_-MeOH 97.5:2.5) and E_4 _(CHCl_3_-MeOH 95:5 to 90:10) were repeatedly filtered through Sephadex LH-20 (CHCl_3_-MeOH 7:3) to yield artonin E C_25_H_24_O_7 _(**4**; 10.0 mg, m/z 436.14; yellow crystals, m.p.: 255-257°C) [13] and 2-[(3,5-dihydroxy)-(Z)-4-(3-methylbut-1-enyl)phenyl]benzofuran-6-ol C_19_H_18_O_4 _(**5**; 12.8 mg; m/z 310.0; yellow oil) [14]. The chemical structures of the isolated compounds are illustrated in Figure 1.

### General procedure

Aluminum sheet pre-coated with silica gel 60 *F*254 nm (Merck) was used for thin layer chromatography; The spots were visualized using both ultraviolet light (254 and 366 nm) and 50% H_2_SO_4 _spray reagent. NMR spectra were recorded on a Bruker Avance 300 at 300 MHz (^1^H) and 75 MHz and Bruker Avance 600 at 600 MHz (^1^H) and 150 MHz (^13^C), with the residual solvent peaks as internal references. The melting point (m.p.) were determined using a Kofler microhot stage apparatus. Mass spectra were recorded with API QSTAR pulsar mass spectrometer. The structures of the compounds were confirmed by comparing with reference data from available literature.

### Antimicrobial assays

#### Microbial strains and culture media

The studied microorganisms included reference strains of *Providencia stuartii, Pseudomonas aeruginosa, Klebsiella pneumoniae, Staphylococcus aureus, Salmonella typhi, Escherichia coli, Candida albicans *obtained from the American Type Culture Collection. They were maintained on agar slant at 4°C and sub-cultured on a fresh appropriate agar plates 24 h prior to any antimicrobial test. Nutrient Agar and Sabouraud Glucose Agar were used for the activation of bacteria and fungi respectively. The Mueller Hinton Broth (MHB) was used for the MIC and MMC determinations. The Mueller Hinton Agar (MHA) was also used for the determination of the MMC on these species [15].

#### Chemicals for antimicrobial assay

Chloramphenicol (Sigma-Aldrich, St. Quentin Fallavier, France) and Nystatin (Sigma-Aldrich) were used as reference antibiotics (RA) respectively against bacteria and *Candida albicans*. *p*-Iodonitrotetrazolium chloride (INT, Sigma-Aldrich) was used as microbial growth indicator [16,17].

#### MIC and MMC determinations

The MIC determinations on bacteria and *C. albicans *were conducted using rapid INT colorimetric assay according to described methods [16,17] with some modifications. Briefly, the test sample was first of all dissolved in 10% (v/v) DMSO/MHB to give a final concentration of 512 μg/ml and serially diluted twofold to obtain concentration ranges. 100 μl of each concentration was added in a well (96-well microplate) containing 95 μl of MHB and 5 μl of inoculum (standardized at 1.5 × 10^6 ^CFU/ml by adjusting the optical density to 0.1 at 600 nm SHIMADZU UV-120-01 spectrophotometer) [18]. The final concentration of DMSO in the well was less than 3% (preliminary analyses with 3% (v/v) DMSO do not alter the growth of the test organisms). The negative control well consisted of 195 μl of MHB and 5 μl of the standard inoculum [19]. The plates were covered with a sterile plate sealer, then agitated to mix the contents of the wells using a plate shaker and incubated at 37°C for 24 h. The assay was repeated three times in triplicate. The MIC of samples was detected following addition (40 μl) of 0.2 mg/ml *p*-iodonitrotetrazolium chloride and incubation at 37°C for 30 min [16,17]. Viable microorganisms reduced the yellow dye to a pink colour. MIC was defined as the lowest sample concentration that prevented this change and exhibited complete inhibition of bacterial growth. For the determination of MMC, a portion of liquid (5 μl) from each well that showed no change in colour was plated on MHA and incubated at 37°C for 24 h. The lowest concentration that yielded no growth after this sub-culturing was taken as the MMC [20].

## Results and discussion

The structures of the isolated compounds were established using spectroscopic analysis, especially, NMR spectra in conjunction with 2D experiments, COSY, HMQC, HMBC, and direct comparison with published information and with authentic specimens obtained in our research group for some cases. The compounds isolated from the stem bark of *A. communis *(Figure 1) were identified as peruvianursenyl acetate C (**1**), α-amyrenol or viminalol (**2**), sitosterol 3-O- *ß-D*-glucopyranoside (**3**), artonin E (**4**) and 2-[(3,5-dihydroxy)-(Z)-4-(3-methylbut-1-enyl)phenyl]benzofuran-6-ol (**5**). Numbers of terpenoids isolated from *A. communis *such as compounds **2 **and **3 **are ubiquitous in plant kingdom. The flavonoid, Artonin E was previously reported in other *Artocapus species, A. kemando*, *A. nobilis *and *A. rigida *[13,21,22] meanwhile the arylbenzofuran, 2-[(3,5-dihydroxy)-(Z)-4-(3-methylbut-1-enyl)phenyl]benzofuran-6-ol was reported in *Artocarpus heterophyllus *[23]. In the present work, the crude extract as well as most of the compounds isolated from the bark of *A. communis *were tested for their antibacterial activities and against *C. albicans*. The results are reported in Tables 1 and 2.

**Table 1:** MIC (μg/ml) of the crude extract, compounds isolated from the bark of *Artocarpus communis *and reference antibiotics on the studied microbial species.

**Tested samples**^**a**^	**Microorganisms, strains and MIC (μg/ml)**^**b**^							
	
						*E. coli*		
							
	*P. stuartii*	*P. aeruginosa*	*K. pneumoniae*	*S.aureus *ATCC25922	*S. typhi *ATCC6539			*C. albicans*
	ATCC29916	PA01	ATCC11296			AG100	ATCC 8739	W3100
ACB	256	256	128	64	128	256	64	128
**1**	-	-	256	512	-	-	-	512
**2**	-	-	512	512	-	-	512	-
**4**	512	32	128	256	64	512	64	512
**5**	128	32	256	256	64	64	32	128
**RA**	32	64	4	4	4	4	4	16

^a^The tested samples were the methanol extract from the bark of *Artocarpus communis *(ACB), isolated compounds, **1**: peruvianursenyl acetate C; **2**: α-amyrenol or viminalol; **3**: sitosterol 3-O- *ß-D*-glucopyranoside; **4**: artonin E; **5**: 2-[(3,5-dihydroxy)-(Z)-4-(3-methylbut-1-enyl)phenyl]benzofuran-6-ol and chloramphenicol (for bacteria) and nystatin (for *C. albicans*) used as the reference antibiotics (RA);

^b^The tested microorganisms were *Providencia stuartii (P. stuartii); Pseudomonas aeruginosa (P. aeruginosa); Klebsiella pneumoniae (K. pneumoniae); Staphylococcus aureus (S. aureus); Salmonella typhi (S. typhi); Escherichia coli (E. coli); Candida albicans (C. albicans)*. (-): MIC >512 μg/ml; (nd): not determined

The MIC results (Table 1) indicated that the crude extract (ACB) as well as compounds **4 **and **5 **inhibited the growth of all tested microbial species. All other compounds showed selective activities, their inhibitory effects being noted on 3 of the 8 (37.5%) tested organisms for compound **1 **and **2**. The lowest MIC value (64 μg/ml) for the crude extract was recorded on two of the tested microbial species namely *S. aureus *and *E. coli *ATCC8739. Phytochemicals are routinely classified as antimicrobials on the basis of susceptibility tests that produce MIC) in the range of 100 to 1000 mg/mL [24]. Their activity is considered to be significant if MICvalues are below 100 μg/ml for crude extract and 10 μg/ml for pure compounds [25]. Therefore, the activity recorded herein can be considered as important, when considering the cutoff point 100 μg/ml required for MIC values of plant extracts with significant activity [25]. Nevertheless, moderate activities [25] were recorded with compounds **4 **and **5 **on three (37.5%) and four (50%) of the studied microorganisms respectively. *P. aeruginosa *is an important nosocomial pathogen highly resistant to commonly used antibiotics, causing a wide spectrum of infections and leading to substantial morbidity and mortality [26]. The lowest MIC value of 32 μg/ml was recorded with compounds **4 **and **5 **on *P. aeruginosa *and compound **5 **on *E. coli *ATCC8739, highlighting some medicinal potential for the two compounds, as the activity on *P. aeruginosa *was better than that of chloramphenicol.

However if considered a more flexible stringent criteria indicating that extracts having activities with MIC values below 8 mg/ml [27] are considered to possess some antimicrobial activity and natural products with MIC values below 1 mg/ml are considered noteworthy [28,29], the overall activity recorded therefore with the extracts, compounds **4**, and **5 **could be considered as important, highlighting the antimicrobial potency of *A. communis*. However, the tested samples were less active than chloramphenicol and nystatin used as reference antibiotic on most of the microbial strains. The results of Table 2 showed detectable MMC values for some of the studied samples on the tested microbial strains. When analysing carefully the MIC and MMC results for the crude extract, compounds **4 **and **5**, it can be noted that MMC/MIC ratios lower than 4 were obtained with these samples on most of the tested microbial species, suggesting that a killing effects could be expected [30]. However, all MMC values obtained were greater than the MICs. It can also be noted the reference antibiotics were in most of the case more active than all studied samples, except on *P. aeruginosa *PA01 where the MIC values obtained with compounds **4 **and **5 **were two time lower.

**Table 2:** MMC (μg/ml) of the crude extract, compounds isolated from the bark of *Artocarpus communis *and reference antibiotics on the studied microbial species.

**Tested samples**^**a**^	**Microorganisms, strains and MMC (μg/ml)**^**b**^							
						***E. coli***		
							
	***P. stuartii***	***P. aeruginosa***	***K. pneumoniae***	***S.aureus *ATCC25922**	***S. typhi *ATCC6539**			***C. albicans***

	ATCC29916	PA01	ATCC11296			AG100	ATCC 8739	W3100
ACB	>512	512	256	256	256	512	256	256
**1**	-	-	256	>512	>512	-	-	>512
**2**	-	-	>512	>512	-	-	>512	nd
**4**	>512	128	256	512	128	>512	128	>512
**5**	256	64	512	512	128	256	64	256
**RA**	64	128	8	8	8	8	8	32

^a^The tested samples were the methanol extract from the bark of *Artocarpus communis *(ACB), isolated compounds, **1**: peruvianursenyl acetate C; **2**: α-amyrenol or viminalol; **3**: sitosterol 3-O- *ß-D*-glucopyranoside; **4**: artonin E; **5**: 2-[(3,5-dihydroxy)-(Z)-4-(3-methylbut-1-enyl)phenyl]benzofuran-6-ol and chloramphenicol (for bacteria) and nystatin (for *C. albicans*) used as the reference antibiotics (RA);

^b^The tested microorganisms were *Providencia stuartii (P. stuartii); Pseudomonas aeruginosa (P. aeruginosa); Klebsiella pneumoniae (K. pneumoniae); Staphylococcus aureus (S. aureus); Salmonella typhi (S. typhi); Escherichia coli (E. coli); Candida albicans (C. albicans)*. (-): MIC >512 μg/ml; (nd): not determined

To the best of our knowledge, the antibacterial and anti-candicidal activities of the bark extract of *A. communis *as well as that of compounds **4**, and **5 **are being reported for the first time. However, the antimicrobial activity of this plant might be due to the presence of both antibacterial and anticandicidal compounds as demonstrated in the present study. The antimicrobial activity of sitosterol-3-*O-β-D*-glucopyranoside (compound **3**) was reported [31,32], and this compound was not tested again in the present work. It's activities were moderate, but sitosterol-3-*O-β-D*-glucopyranoside as well as the tested compounds might contribute to the overall activity observed with the extract of *A. communis*.

## Conclusion

Finally, the present investigation provides supportive data for the use of *A. communis *as well as some of its constituents for the treatment of infections associated with the studied microorganisms. However, this will be confirmed with further pharmacological (*in vivo *activity, bioavailability) and toxicological studies (acute and subacute toxicities using animal models).

## Competing interests

The authors declare that they have no competing interests.

## Authors' contributions

VK and PYA carried out the study and wrote the manuscript; GDWFK, BMA and BTN supervised the work and the manuscript writting. JPD and AGW contributed to the manuscript corrections and editing. All authors read and approved the final manuscript.

## Pre-publication history

The pre-publication history for this paper can be accessed here:

http://www.biomedcentral.com/1472-6882/11/42/prepub
